# Poly[diaqua­(μ_5_-pyridine-3,5-dicarboxyl­ato)strontium]

**DOI:** 10.1107/S1600536812023379

**Published:** 2012-05-31

**Authors:** Dan Li, Chaowen Duan

**Affiliations:** a School of Media Communications, Linyi University, Linyi 276000, People’s Republic of China; b School of Chemistry and Chemical Engineering, Linyi University, Linyi 276000, People’s Republic of China

## Abstract

In the structure of the title compound, [Sr(C_7_H_3_NO_4_)(H_2_O)_2_]_*n*_, the Sr^II^ cation is eight-coordinated in form of a distorted dodeca­hedron by two water O atoms and by five O atoms and one N atom from five pyridine-3,5-dicarboxyl­ate anions. The bridging mode of the anions leads to the formation of a layered network parallel to (100). O—H⋯O hydrogen bonding between the coordinating water mol­ecules and the carboxyl­ate groups of adjacent layers consolidates the crystal packing. Weak C—H⋯O inter­actions are also observed.

## Related literature

For related structures with pyridine-3,5-dicarboxyl­ato ligands, see: Aghabozorg *et al.* (2008 ▶); Dang *et al.* (2010 ▶); Du *et al.* (2009 ▶); Lv *et al.* (2010 ▶); Wu *et al.* (2008 ▶); Yao *et al.* (2010 ▶).
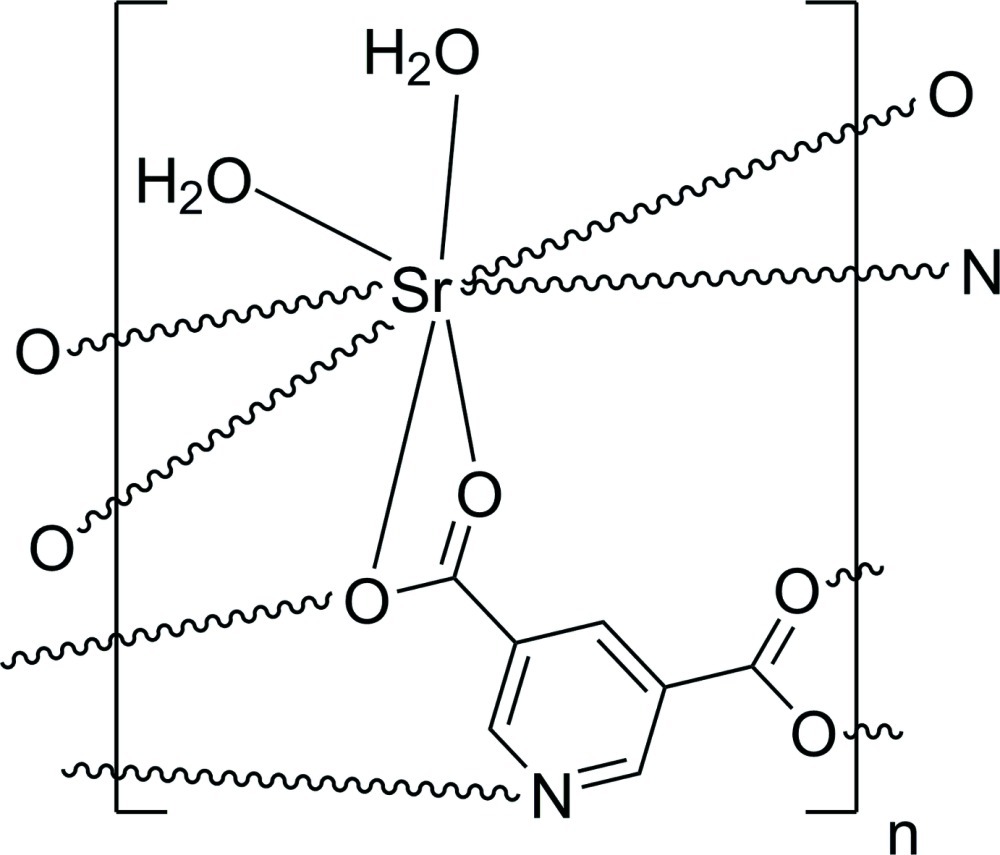


## Experimental

### 

#### Crystal data

[Sr(C_7_H_3_NO_4_)(H_2_O)_2_]
*M* *_r_* = 288.76
Triclinic, 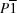

*a* = 7.9098 (4) Å
*b* = 8.0028 (4) Å
*c* = 8.0864 (5) Å
α = 88.620 (2)°
β = 71.270 (2)°
γ = 72.030 (2)°
*V* = 459.52 (4) Å^3^
*Z* = 2
Mo *K*α radiation
μ = 5.88 mm^−1^
*T* = 293 K
0.30 × 0.17 × 0.16 mm

#### Data collection

Bruker APEXII CCD diffractometer
Absorption correction: multi-scan (*SADABS*; Bruker, 2005 ▶) *T*_min_ = 0.272, *T*_max_ = 0.453
8322 measured reflections
2305 independent reflections
2226 reflections with *I* > 2σ(*I*)
*R*_int_ = 0.022

#### Refinement

*R*[*F* ^2^ > 2σ(*F* ^2^)] = 0.017
*wR*(*F* ^2^) = 0.046
*S* = 1.08
2305 reflections
136 parameters
H-atom parameters constrained
Δρ_max_ = 0.38 e Å^−3^
Δρ_min_ = −0.51 e Å^−3^

### 

Data collection: *APEX2* (Bruker, 2005 ▶); cell refinement: *SAINT* (Bruker, 2005 ▶); data reduction: *SAINT*; program(s) used to solve structure: *SHELXS97* (Sheldrick, 2008 ▶); program(s) used to refine structure: *SHELXL97* (Sheldrick, 2008 ▶); molecular graphics: *XP* in *SHELXTL* (Sheldrick, 2008 ▶) and *DIAMOND* (Brandenburg, 2012 ▶); software used to prepare material for publication: *SHELXTL*.

## Supplementary Material

Crystal structure: contains datablock(s) I, global. DOI: 10.1107/S1600536812023379/wm2630sup1.cif

Structure factors: contains datablock(s) I. DOI: 10.1107/S1600536812023379/wm2630Isup2.hkl

Additional supplementary materials:  crystallographic information; 3D view; checkCIF report

## Figures and Tables

**Table 1:** Hydrogen-bond geometry (Å, °)

*D*—H⋯*A*	*D*—H	H⋯*A*	*D*⋯*A*	*D*—H⋯*A*
O5—H5*A*⋯O4^i^	0.85	2.01	2.8046 (17)	155
O5—H5*B*⋯O3^ii^	0.85	2.05	2.8718 (18)	163
O6—H6*A*⋯O2^iii^	0.85	1.87	2.7135 (17)	172
O6—H6*B*⋯O5^iv^	0.86	2.03	2.848 (2)	160
C1—H1⋯O3^ii^	0.93	2.37	3.286 (2)	169

Symmetry codes: (i) 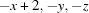; (ii) 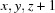; (iii) 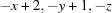; (iv) 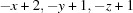.

**Fig. 1.**
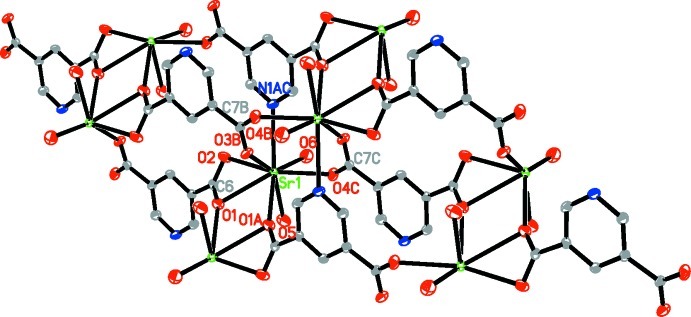
The molecular structure of compound (I), with displacement ellipsoids drawn at the 50% probability level [H-atoms have been omitted for clarity; symmetry codes: (A) -x + 1, -y + 1, -z + 1; (B) -x + 1, -y + 1, -z; (C) x, y + 1, z + 1; (AC) x, y + 1, z.

**Fig. 2.**
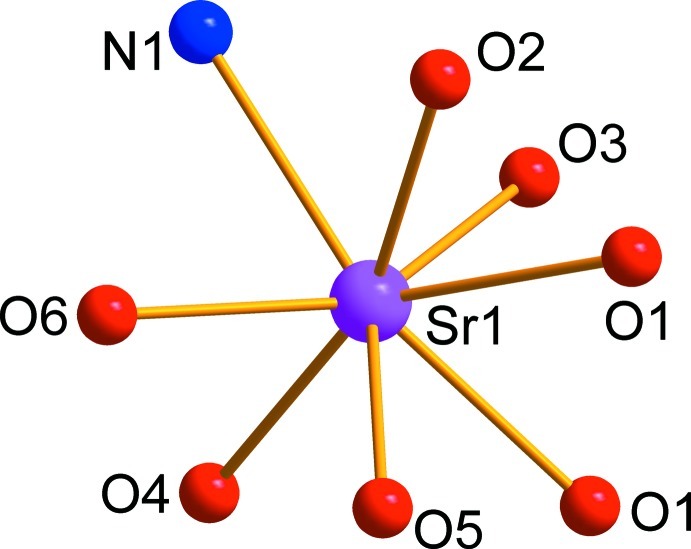
A view of the distorted dodecahedral environment around the SrII atom in (I).

**Fig. 3.**
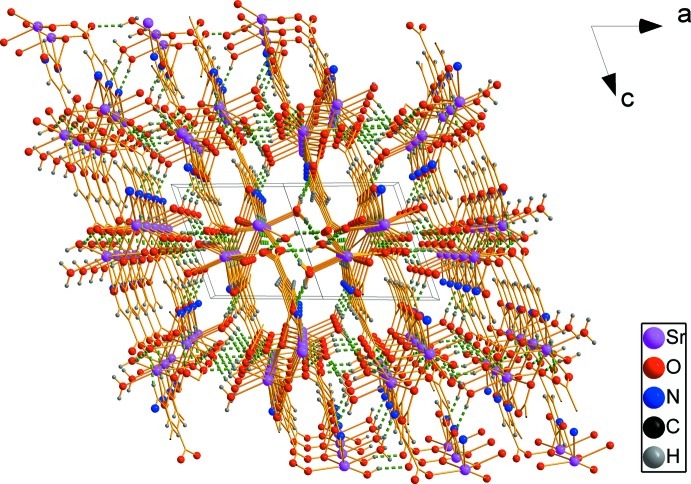
A view down the b axis of the crystal packing of compound (I). Hydrogen bonds are shown as dashed lines.

## supplementary crystallographic information

### Comment

Pyridine-3,5-dicarboxylic acid (py-3,5-dc) is an interesting ligand because it can act in a multidentate fashion and also can participate in hydrogen bonding interactions with its N and O acceptors. Some polymeric complexes of this ligand with 3*d* metals (Dang *et al.*, 2010; Lv *et al.*, 2010; Du *et al.*, 2009) and with mixed 3*d*-4*f* metals (Yao *et al.*, 2010; Wu *et al.*, 2008) have been published. A related strontium complex with a chain-structure has been reported by Aghabozorg *et al.* (2008). Here we report the layered structure of another polymeric strontium complex, [Sr(C_7_H_3_NO_4_)(H_2_O)_2_]_n_, (I).

The coordination number of the cation is eight, with bonds to seven O and one N atoms from five py-3,5-dc anions (Fig. 1). The corresponding geometry is distorted dodecahedral (Fig. 2). The Sr—O distances are in the range of 2.5287 (13)–2.7232 (12) Å, and the Sr—N bond is the longest of the coordination polyhedron with a length of 2.8064 (14) Å. The carboxylate O1 atom links two cations to form a rhombic binuclear Sr_2_O_2_ unit. These units are further connected by carboxylate groups and N atoms of symmetry-related ligands to form a two-dimensional network parallel to (100). Atoms O2, O3 and O4 from these ligands are acceptor atoms for hydrogen bonding with coordinating water molecules of adjacent ligands as donor atoms, leading to a three-dimensional set-up of the structure (Table 1 and Fig. 3). Weak C—H···O interactions are also observed.

### Experimental

A mixture of 3,5-H_2_pdc (0.0330 g, 0.2 mmol), SrCl_2_^.^6H_2_O (0.0539 g, 0.2 mmol), imidazole (0.0320 g, 0.47 mmol), C_2_H_5_OH / H_2_O = 1: 2 (3 ml) was placed in a Pyrex-tube (8 ml). The tube was heated for 4 days at 393 K under autogenous pressure. Colourless rod-shaped crystals were obtained. Anal. Calc. for C_7_H_7_NO_6_Sr: C, 29.12; N, 4.85; H, 2.44. Found: C, 28.65; N, 4.60; H, 2.35%. IR (KBr, cm^-1^): 3490 br, 1610 s, 1552 s, 1455 s, 1421 s, 1385 s, 1311 s, 1132 s, 1030 s, 941 s, 827 s, 782 s, 739 s, 651 s, 570 s, 439 s.

### Refinement

All hydrogen atoms were located in difference Fourier maps. The water H-atoms were restrained to bond lengths of O—H = 0.85–0.86 Å and with a common *U*_iso_ parameter of 0.028 Å^2^ for the H atoms. The C-bound H-atoms were included in calculated positions and treated as riding atoms with C—H = 0.95 Å and *U*_iso_(H) = 1.2 *U*_eq_(C).

### Crystal data

**Table:**

[Sr(C_7_H_3_NO_4_)(H_2_O)_2_]	*Z* = 2
*M**_r_* = 288.76	*F*(000) = 284
Triclinic, *P*1	*D*_x_ = 2.087 Mg m^−^^3^
Hall symbol: -P 1	Mo *K*α radiation, λ = 0.71073 Å
*a* = 7.9098 (4) Å	Cell parameters from 3510 reflections
*b* = 8.0028 (4) Å	θ = 2.7–25.3°
*c* = 8.0864 (5) Å	µ = 5.88 mm^−^^1^
α = 88.620 (2)°	*T* = 293 K
β = 71.270 (2)°	Block, colourless
γ = 72.030 (2)°	0.30 × 0.17 × 0.16 mm
*V* = 459.52 (4) Å^3^	

### Data collection

**Table:**

Bruker APEXII CCD diffractometer	2305 independent reflections
Radiation source: fine-focus sealed tube	2226 reflections with *I* > 2σ(*I*)
Graphite monochromator	*R*_int_ = 0.022
φ and ω scans	θ_max_ = 28.4°, θ_min_ = 2.7°
Absorption correction: multi-scan (*SADABS*; Bruker, 2005)	*h* = −10→10
*T*_min_ = 0.272, *T*_max_ = 0.453	*k* = −10→10
8322 measured reflections	*l* = −9→10

### Refinement

**Table:**

Refinement on *F*^2^	Primary atom site location: structure-invariant direct methods
Least-squares matrix: full	Secondary atom site location: difference Fourier map
*R*[*F*^2^ > 2σ(*F*^2^)] = 0.017	Hydrogen site location: inferred from neighbouring sites
*wR*(*F*^2^) = 0.046	H-atom parameters constrained
*S* = 1.08	*w* = 1/[σ^2^(*F*_o_^2^) + (0.0269*P*)^2^ + 0.1887*P*] where *P* = (*F*_o_^2^ + 2*F*_c_^2^)/3
2305 reflections	(Δ/σ)_max_ = 0.001
136 parameters	Δρ_max_ = 0.38 e Å^−^^3^
0 restraints	Δρ_min_ = −0.51 e Å^−^^3^

### Special details

**Table:**

Geometry. All e.s.d.'s (except the e.s.d. in the dihedral angle between two l.s. planes) are estimated using the full covariance matrix. The cell e.s.d.'s are taken into account individually in the estimation of e.s.d.'s in distances, angles and torsion angles; correlations between e.s.d.'s in cell parameters are only used when they are defined by crystal symmetry. An approximate (isotropic) treatment of cell e.s.d.'s is used for estimating e.s.d.'s involving l.s. planes.
Refinement. Refinement of *F*^2^ against ALL reflections. The weighted *R*-factor *wR* and goodness of fit *S* are based on *F*^2^, conventional *R*-factors *R* are based on *F*, with *F* set to zero for negative *F*^2^. The threshold expression of *F*^2^ > σ(*F*^2^) is used only for calculating *R*-factors(gt) *etc*. and is not relevant to the choice of reflections for refinement. *R*-factors based on *F*^2^ are statistically about twice as large as those based on *F*, and *R*- factors based on ALL data will be even larger.

### Fractional atomic coordinates and isotropic or equivalent isotropic displacement parameters (Å^2^)

**Table:**

	*x*	*y*	*z*	*U*_iso_*/*U*_eq_	
Sr1	0.685514 (18)	0.654700 (16)	0.373999 (17)	0.01302 (5)	
O1	0.53412 (17)	0.40183 (16)	0.33347 (15)	0.0220 (2)	
O3	0.63315 (17)	0.12206 (15)	−0.40559 (15)	0.0206 (2)	
O2	0.7094 (2)	0.47815 (16)	0.09101 (16)	0.0260 (3)	
O4	0.76724 (18)	−0.16816 (16)	−0.41598 (16)	0.0215 (2)	
O5	0.85222 (17)	0.35498 (17)	0.47402 (19)	0.0288 (3)	
O6	1.03912 (18)	0.5746 (2)	0.23839 (17)	0.0303 (3)	
N1	0.7457 (2)	−0.11960 (18)	0.10565 (18)	0.0204 (3)	
C7	0.7063 (2)	−0.0153 (2)	−0.34150 (19)	0.0143 (3)	
C4	0.7184 (2)	0.0071 (2)	−0.16144 (19)	0.0146 (3)	
C3	0.6819 (2)	0.1745 (2)	−0.0860 (2)	0.0150 (3)	
H3	0.6607	0.2725	−0.1498	0.018*	
C2	0.6775 (2)	0.1931 (2)	0.0855 (2)	0.0148 (3)	
C5	0.7511 (2)	−0.1354 (2)	−0.0614 (2)	0.0182 (3)	
H5	0.7781	−0.2475	−0.1125	0.022*	
C6	0.6365 (2)	0.3698 (2)	0.1760 (2)	0.0155 (3)	
C1	0.7093 (2)	0.0429 (2)	0.1755 (2)	0.0189 (3)	
H1	0.7051	0.0559	0.2909	0.023*	
H6A	1.1093	0.5659	0.1327	0.028*	
H5A	0.9661	0.2895	0.4265	0.028*	
H5B	0.7950	0.2793	0.4868	0.028*	
H6B	1.0903	0.5704	0.3176	0.028*	

### Atomic displacement parameters (Å^2^)

**Table:**

	*U*^11^	*U*^22^	*U*^33^	*U*^12^	*U*^13^	*U*^23^
Sr1	0.01678 (8)	0.01181 (8)	0.01109 (8)	−0.00511 (5)	−0.00483 (5)	0.00017 (5)
O1	0.0290 (6)	0.0226 (6)	0.0127 (5)	−0.0120 (5)	−0.0006 (5)	−0.0034 (4)
O3	0.0265 (6)	0.0195 (6)	0.0153 (5)	−0.0035 (5)	−0.0100 (5)	0.0024 (4)
O2	0.0417 (8)	0.0208 (6)	0.0155 (6)	−0.0187 (5)	−0.0006 (5)	−0.0019 (5)
O4	0.0266 (6)	0.0181 (6)	0.0196 (6)	−0.0042 (5)	−0.0097 (5)	−0.0059 (5)
O5	0.0194 (6)	0.0245 (6)	0.0418 (8)	−0.0066 (5)	−0.0101 (6)	0.0104 (6)
O6	0.0222 (6)	0.0457 (8)	0.0198 (6)	−0.0126 (6)	−0.0007 (5)	0.0007 (6)
N1	0.0317 (8)	0.0162 (6)	0.0153 (6)	−0.0076 (5)	−0.0104 (6)	0.0037 (5)
C7	0.0158 (7)	0.0163 (7)	0.0110 (7)	−0.0058 (5)	−0.0040 (5)	−0.0006 (5)
C4	0.0177 (7)	0.0141 (7)	0.0120 (7)	−0.0048 (5)	−0.0052 (5)	−0.0009 (5)
C3	0.0202 (7)	0.0128 (7)	0.0131 (7)	−0.0065 (5)	−0.0056 (5)	0.0013 (5)
C2	0.0195 (7)	0.0147 (7)	0.0117 (7)	−0.0077 (5)	−0.0044 (5)	−0.0016 (5)
C5	0.0268 (8)	0.0127 (7)	0.0161 (7)	−0.0060 (6)	−0.0084 (6)	−0.0004 (6)
C6	0.0201 (7)	0.0156 (7)	0.0125 (7)	−0.0070 (6)	−0.0063 (6)	−0.0014 (6)
C1	0.0273 (8)	0.0196 (8)	0.0121 (7)	−0.0093 (6)	−0.0079 (6)	0.0016 (6)

### Geometric parameters (Å, º)

**Table:**

Sr1—O6	2.5287 (13)	O5—H5A	0.8542
Sr1—O3^i^	2.5377 (12)	O5—H5B	0.8477
Sr1—O1^ii^	2.5715 (12)	O6—H6A	0.8468
Sr1—O4^iii^	2.5881 (12)	O6—H6B	0.8563
Sr1—O5	2.6050 (13)	N1—C1	1.339 (2)
Sr1—O2	2.6423 (12)	N1—C5	1.345 (2)
Sr1—O1	2.7232 (12)	N1—Sr1^vi^	2.8065 (14)
Sr1—N1^iv^	2.8064 (14)	C7—C4	1.508 (2)
Sr1—C6	3.0051 (15)	C4—C5	1.390 (2)
Sr1—H6B	2.9429	C4—C3	1.391 (2)
O1—C6	1.2520 (19)	C3—C2	1.387 (2)
O1—Sr1^ii^	2.5715 (12)	C3—H3	0.9300
O3—C7	1.2590 (19)	C2—C1	1.388 (2)
O3—Sr1^i^	2.5377 (12)	C2—C6	1.502 (2)
O2—C6	1.258 (2)	C5—H5	0.9300
O4—C7	1.2554 (19)	C1—H1	0.9300
O4—Sr1^v^	2.5881 (12)		
			
O6—Sr1—O3^i^	146.80 (4)	O1^ii^—Sr1—H6B	119.9
O6—Sr1—O1^ii^	133.77 (4)	O4^iii^—Sr1—H6B	65.0
O3^i^—Sr1—O1^ii^	75.71 (4)	O5—Sr1—H6B	63.0
O6—Sr1—O4^iii^	77.95 (4)	O2—Sr1—H6B	98.6
O3^i^—Sr1—O4^iii^	95.67 (4)	O1—Sr1—H6B	121.6
O1^ii^—Sr1—O4^iii^	81.28 (4)	N1^iv^—Sr1—H6B	85.2
O6—Sr1—O5	69.37 (4)	C6—Sr1—H6B	107.7
O3^i^—Sr1—O5	143.83 (4)	Sr1^ii^—Sr1—H6B	128.1
O1^ii^—Sr1—O5	70.70 (4)	C6—O1—Sr1^ii^	156.53 (11)
O4^iii^—Sr1—O5	92.21 (4)	C6—O1—Sr1	90.40 (9)
O6—Sr1—O2	84.52 (4)	Sr1^ii^—O1—Sr1	112.09 (4)
O3^i^—Sr1—O2	95.32 (4)	C7—O3—Sr1^i^	140.72 (10)
O1^ii^—Sr1—O2	116.27 (4)	C6—O2—Sr1	94.03 (9)
O4^iii^—Sr1—O2	161.21 (4)	C7—O4—Sr1^v^	137.91 (10)
O5—Sr1—O2	87.86 (4)	Sr1—O5—H5A	127.0
O6—Sr1—O1	115.98 (4)	Sr1—O5—H5B	115.1
O3^i^—Sr1—O1	86.90 (4)	H5A—O5—H5B	100.7
O1^ii^—Sr1—O1	67.91 (4)	Sr1—O6—H6A	131.4
O4^iii^—Sr1—O1	147.46 (4)	Sr1—O6—H6B	110.7
O5—Sr1—O1	68.48 (4)	H6A—O6—H6B	117.3
O2—Sr1—O1	48.48 (4)	C1—N1—C5	117.16 (14)
O6—Sr1—N1^iv^	74.58 (5)	C1—N1—Sr1^vi^	109.60 (10)
O3^i^—Sr1—N1^iv^	73.32 (4)	C5—N1—Sr1^vi^	130.26 (11)
O1^ii^—Sr1—N1^iv^	147.89 (4)	O4—C7—O3	124.65 (14)
O4^iii^—Sr1—N1^iv^	93.47 (4)	O4—C7—C4	118.35 (14)
O5—Sr1—N1^iv^	141.38 (4)	O3—C7—C4	117.00 (13)
O2—Sr1—N1^iv^	75.20 (4)	C5—C4—C3	118.26 (14)
O1—Sr1—N1^iv^	118.12 (4)	C5—C4—C7	121.27 (14)
O6—Sr1—C6	97.37 (4)	C3—C4—C7	120.27 (13)
O3^i^—Sr1—C6	95.75 (4)	C2—C3—C4	119.12 (14)
O1^ii^—Sr1—C6	92.37 (4)	C2—C3—H3	120.4
O4^iii^—Sr1—C6	165.14 (4)	C4—C3—H3	120.4
O5—Sr1—C6	72.98 (4)	C3—C2—C1	118.28 (14)
O2—Sr1—C6	24.68 (4)	C3—C2—C6	122.02 (14)
O1—Sr1—C6	24.62 (4)	C1—C2—C6	119.69 (13)
N1^iv^—Sr1—C6	98.90 (4)	N1—C5—C4	123.41 (14)
O6—Sr1—Sr1^ii^	132.57 (3)	N1—C5—H5	118.3
O3^i^—Sr1—Sr1^ii^	79.75 (3)	C4—C5—H5	118.3
O1^ii^—Sr1—Sr1^ii^	35.06 (3)	O1—C6—O2	122.85 (14)
O4^iii^—Sr1—Sr1^ii^	115.72 (3)	O1—C6—C2	118.69 (14)
O5—Sr1—Sr1^ii^	65.11 (3)	O2—C6—C2	118.45 (14)
O2—Sr1—Sr1^ii^	81.27 (3)	O1—C6—Sr1	64.98 (8)
O1—Sr1—Sr1^ii^	32.85 (2)	O2—C6—Sr1	61.29 (8)
N1^iv^—Sr1—Sr1^ii^	142.01 (3)	C2—C6—Sr1	159.61 (11)
C6—Sr1—Sr1^ii^	57.36 (3)	N1—C1—C2	123.75 (14)
O6—Sr1—H6B	15.8	N1—C1—H1	118.1
O3^i^—Sr1—H6B	150.4	C2—C1—H1	118.1
			
O6—Sr1—O1—C6	−43.90 (11)	Sr1—O1—C6—O2	−21.19 (17)
O3^i^—Sr1—O1—C6	111.03 (10)	Sr1^ii^—O1—C6—C2	−6.4 (4)
O1^ii^—Sr1—O1—C6	−173.14 (12)	Sr1—O1—C6—C2	157.48 (13)
O4^iii^—Sr1—O1—C6	−153.28 (9)	Sr1^ii^—O1—C6—Sr1	−163.9 (3)
O5—Sr1—O1—C6	−96.05 (10)	Sr1—O2—C6—O1	21.92 (17)
O2—Sr1—O1—C6	11.13 (9)	Sr1—O2—C6—C2	−156.75 (12)
N1^iv^—Sr1—O1—C6	41.81 (11)	C3—C2—C6—O1	139.97 (16)
Sr1^ii^—Sr1—O1—C6	−173.14 (12)	C1—C2—C6—O1	−39.0 (2)
O6—Sr1—O1—Sr1^ii^	129.25 (5)	C3—C2—C6—O2	−41.3 (2)
O3^i^—Sr1—O1—Sr1^ii^	−75.83 (5)	C1—C2—C6—O2	139.73 (16)
O1^ii^—Sr1—O1—Sr1^ii^	0.0	C3—C2—C6—Sr1	−125.0 (3)
O4^iii^—Sr1—O1—Sr1^ii^	19.86 (10)	C1—C2—C6—Sr1	56.1 (4)
O5—Sr1—O1—Sr1^ii^	77.09 (5)	O6—Sr1—C6—O1	141.06 (10)
O2—Sr1—O1—Sr1^ii^	−175.73 (8)	O3^i^—Sr1—C6—O1	−69.51 (10)
N1^iv^—Sr1—O1—Sr1^ii^	−145.05 (5)	O1^ii^—Sr1—C6—O1	6.36 (11)
C6—Sr1—O1—Sr1^ii^	173.14 (12)	O4^iii^—Sr1—C6—O1	70.52 (19)
O6—Sr1—O2—C6	121.16 (11)	O5—Sr1—C6—O1	75.34 (10)
O3^i^—Sr1—O2—C6	−92.19 (10)	O2—Sr1—C6—O1	−159.75 (16)
O1^ii^—Sr1—O2—C6	−15.52 (11)	N1^iv^—Sr1—C6—O1	−143.48 (9)
O4^iii^—Sr1—O2—C6	142.24 (12)	Sr1^ii^—Sr1—C6—O1	4.41 (8)
O5—Sr1—O2—C6	51.69 (10)	O6—Sr1—C6—O2	−59.19 (11)
O1—Sr1—O2—C6	−11.10 (9)	O3^i^—Sr1—C6—O2	90.24 (10)
N1^iv^—Sr1—O2—C6	−163.36 (11)	O1^ii^—Sr1—C6—O2	166.11 (10)
Sr1^ii^—Sr1—O2—C6	−13.45 (10)	O4^iii^—Sr1—C6—O2	−129.73 (16)
Sr1^v^—O4—C7—O3	−33.4 (3)	O5—Sr1—C6—O2	−124.91 (11)
Sr1^v^—O4—C7—C4	145.77 (12)	O1—Sr1—C6—O2	159.75 (16)
Sr1^i^—O3—C7—O4	105.41 (18)	N1^iv^—Sr1—C6—O2	16.27 (11)
Sr1^i^—O3—C7—C4	−73.8 (2)	Sr1^ii^—Sr1—C6—O2	164.16 (11)
O4—C7—C4—C5	−15.8 (2)	O6—Sr1—C6—C2	35.7 (3)
O3—C7—C4—C5	163.49 (15)	O3^i^—Sr1—C6—C2	−174.9 (3)
O4—C7—C4—C3	169.42 (14)	O1^ii^—Sr1—C6—C2	−99.0 (3)
O3—C7—C4—C3	−11.3 (2)	O4^iii^—Sr1—C6—C2	−34.8 (4)
C5—C4—C3—C2	−0.7 (2)	O5—Sr1—C6—C2	−30.0 (3)
C7—C4—C3—C2	174.30 (14)	O2—Sr1—C6—C2	94.9 (3)
C4—C3—C2—C1	−0.2 (2)	O1—Sr1—C6—C2	−105.4 (3)
C4—C3—C2—C6	−179.19 (14)	N1^iv^—Sr1—C6—C2	111.2 (3)
C1—N1—C5—C4	−1.0 (3)	Sr1^ii^—Sr1—C6—C2	−100.9 (3)
Sr1^vi^—N1—C5—C4	157.24 (12)	C5—N1—C1—C2	0.0 (3)
C3—C4—C5—N1	1.3 (2)	Sr1^vi^—N1—C1—C2	−162.51 (13)
C7—C4—C5—N1	−173.61 (15)	C3—C2—C1—N1	0.6 (2)
Sr1^ii^—O1—C6—O2	174.94 (19)	C6—C2—C1—N1	179.60 (15)

Symmetry codes: (i) −*x*+1, −*y*+1, −*z*; (ii) −*x*+1, −*y*+1, −*z*+1; (iii) *x*, *y*+1, *z*+1; (iv) *x*, *y*+1, *z*; (v) *x*, *y*−1, *z*−1; (vi) *x*, *y*−1, *z*.

### Hydrogen-bond geometry (Å, º)

**Table:**

*D*—H···*A*	*D*—H	H···*A*	*D*···*A*	*D*—H···*A*
O5—H5*A*···O4^vii^	0.85	2.01	2.8046 (17)	155
O5—H5*B*···O3^viii^	0.85	2.05	2.8718 (18)	163
O6—H6*A*···O2^ix^	0.85	1.87	2.7135 (17)	172
O6—H6*B*···O5^x^	0.86	2.03	2.848 (2)	160
C1—H1···O3^viii^	0.93	2.37	3.286 (2)	169

Symmetry codes: (vii) −*x*+2, −*y*, −*z*; (viii) *x*, *y*, *z*+1; (ix) −*x*+2, −*y*+1, −*z*; (x) −*x*+2, −*y*+1, −*z*+1.
